# Supplementary material for: CoCoScore: context-aware co-occurrence scoring for text mining applications using distant supervision
Source: Bioinformatics. 2019 Jun 14;36(1):264–71. doi: 10.1093/bioinformatics/btz490 (PMC6956794; doi:10.1093/bioinformatics/btz490)
Supplement: btz490_Supplementary_Material [file btz490_supplementary_material.pdf]

Supplementary Material
CoCoScore: Context-aware co-occurrence scoring for text mining applications using distant supervision

Alexander Junge and Lars Juhl Jensen

Disease Systems Biology Program,
Novo Nordisk Foundation Center for Protein Research,
University of Copenhagen,
Blegdamsvej 3B, 2200 Copenhagen N, Denmark

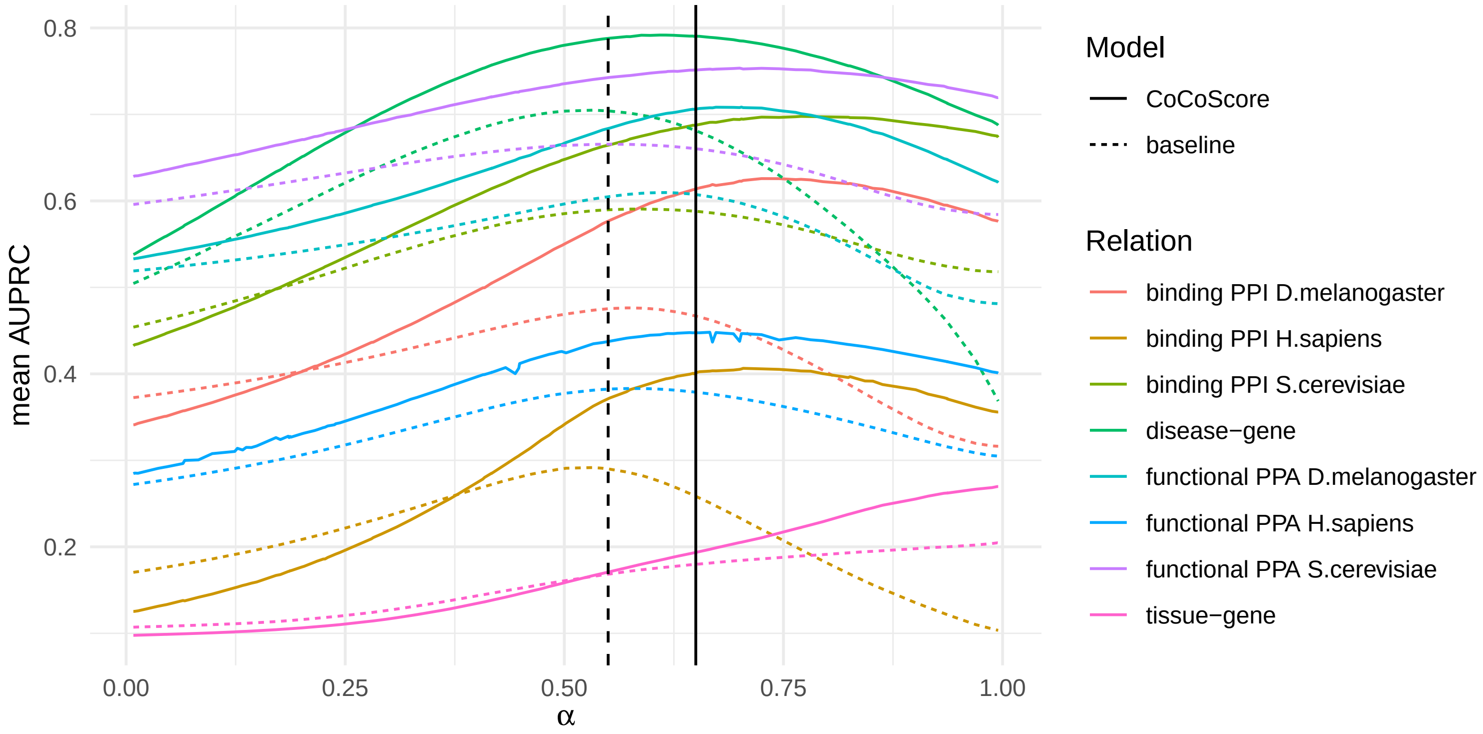

Figure 1: Cross-validation performance for CoCoScore and baseline. Values shown are the mean area under the precision-recall curve (AUPRC) across the three cross-validation sets. α chosen for each data set is depicted as vertical solid or broken lines, respectively. Note that AUPRC is not adjusted to a fixed prior since this adjustment does not affect the choice of α.

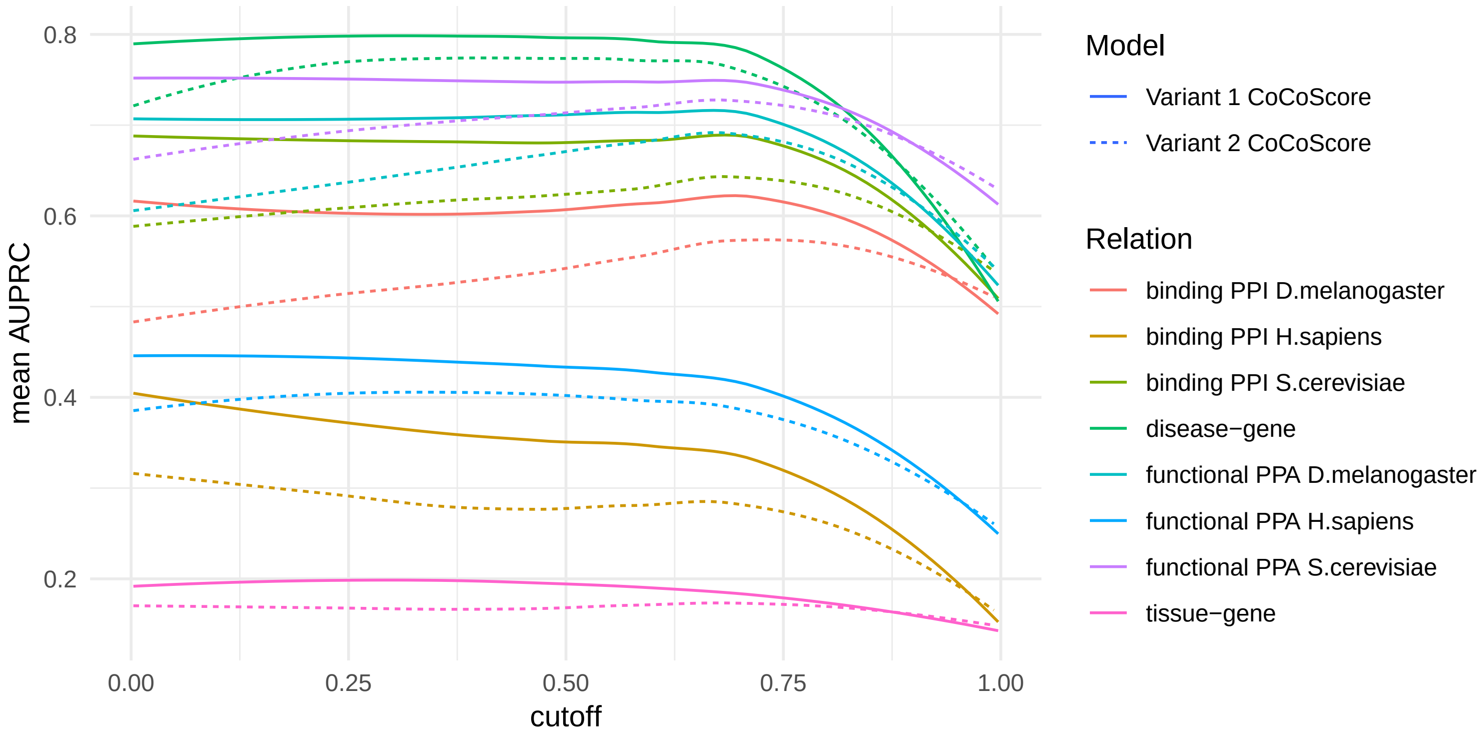

Figure 2: Cross-validation performance for two variants of the CoCoScore model. Compared to the CoCoScore model described in Section 2.3.3 of the main paper and evaluated in Supplementary Figure 1, both variants use the sentence scoring model M as a filter by disregarding sentences with a score lower than a cutoff c. Variant 1 uses $s_k^1(i,j)$ and variant 2 uses $s_k^2(i,j)$ as defined below to score the sentences co-mentioning i and j in each document. The difference between the variants is that variant 2 sets all sentence scores above the cutoff to 1 while variant 1 uses the actual sentence scores in the final co-occurrence scoring. Performance values shown are the mean area under the precision-recall curve (AUPRC) across the three cross-validation sets with α set to 0.65. Note that AUPRC is not adjusted to a fixed prior since this adjustment does not affect the optimal choice of the cutoff c. Variant 1 outperforms variant 2 on all datasets. Variant 1 furthermore achieves the best performance for most (5 out of 8) datasets when the cutoff is 0.0, rendering the cutoff inactive. For the remaining datasets, the AUPRC difference between the optimal choice of the cutoff and a cutoff of 0.0 is negligible (< 0.01). This analysis shows that CoCoScore performs best when the full information from the scoring model M is available in the final co-occurrence scoring step. The final co-occurrence scoring step interprets the sentence score produced by M as the probability that the given sentence states an association and its performance benefits from having access to all sentence scores. Thus, neither variant 1 nor variant 2 were considered for further evaluation and all test set evaluation in the main paper was performed using the CoCoScore model as defined in Section 2.3.3. Note that the cross-validation performance numbers in this Figure should not be compared with the performance on the independent test set shown in the Results section of our main paper.

$$s_k^1(i,j) = \begin{cases} \max\{r_u(i,j) | u \in T_k(i,j) \wedge u \geq c\} & \text{if } i \text{ and } j \text{ are co-mentioned in } k \\ 0 & \text{else} \end{cases}$$

$$s_k^2(i,j) = \begin{cases} 1 & \text{if } i \text{ and } j \text{ are co-mentioned in } k \text{ and } \|\{u \in T_k(i,j) \wedge u \geq c\}\| > 0 \\ 0 & \text{else} \end{cases}$$

Where $\{r_u(i,j) | u \in T_k(i,j) \wedge u \geq c\}$ is the set of scores $\geq$ the cutoff c for sentences co-mentioning i and j in document k as returned by M.

Table 1: AUROC for CoCoScore and baseline model on tissue-gene and disease-gene datasets generated via distant supervision.

method	disease–gene	tissue–gene
CoCoScore	0.98	0.70
baseline	0.96	0.65

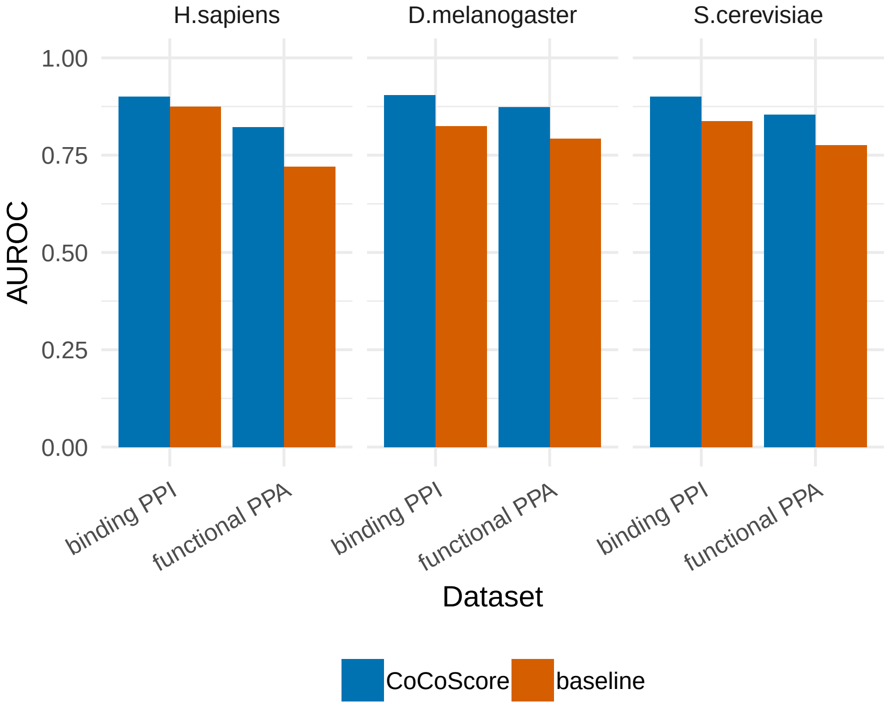

Figure 3: Performance for functional protein–protein associations and physical protein–protein interactions across *H. sapiens*, *D. melanogaster*, and *S. cerevisiae* for both CoCoScore (blue) and the baseline model (red). Performance is depicted as AUROC.

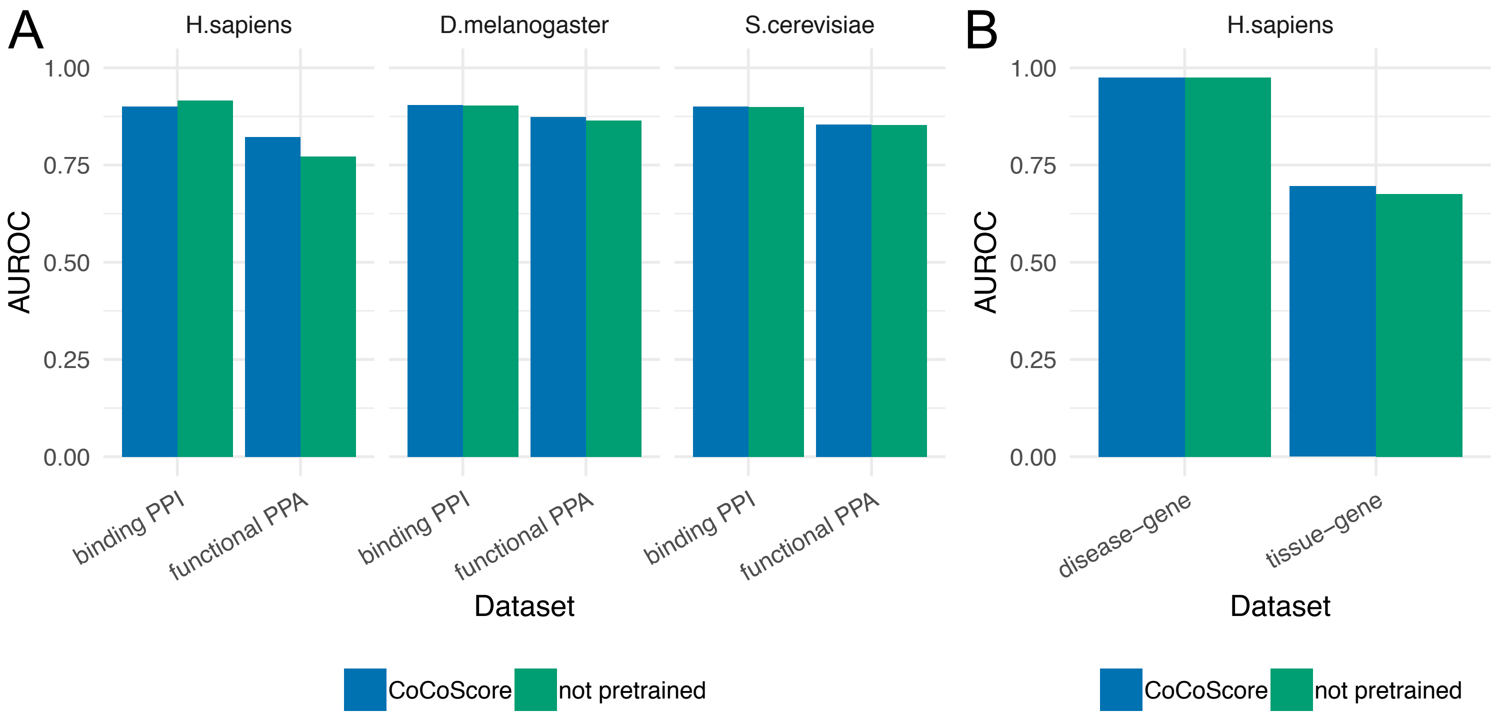

Figure 4: Performance with and without pretrained word embeddings for functional PPA and binding PPI datasts (A) as well as disease–gene and tissue–gene associations (B) for CoCoScore with (blue) and without (green) pre-trained word embeddings. Performance is depicted as AUROC.
